# Indicators of Male Gout Patients' Comorbidities with the Theory on Traditional Chinese Medicine

**DOI:** 10.1155/2018/9679213

**Published:** 2018-12-04

**Authors:** Jianyong Zhang, Siyang Zhang, Jingjing Xie, Li Tang, Xia Qiu, Yuya Xiao, Yanying Zhang, Ertao Jia, Xu Ma, Binbin Wang

**Affiliations:** ^1^Department of Rheumatism, Shenzhen Traditional Chinese Medicine Hospital, Shenzhen, Guangdong, China; ^2^The Fourth Clinical Medical College of Guangzhou University of Chinese Medicine, Guangzhou, China; ^3^Graduate School of Peking Union Medical College, Beijing, China; ^4^Center for Genetics, National Research Institute for Family Planning, Beijing, China

## Abstract

Gout, typically manifesting as acute burning pain and swelling in a joint, has a high frequency of comorbidities. Based on Traditional Chinese Medicine syndrome (TCMS) theory, obstruction of dampness and heat syndrome (ODHS) and intermingled phlegm-stasis blood syndrome (IPSBS) were the two main TCMS subtypes in Chinese suffering from acute gout. In this study, we did a retrospective study enrolling 4,417 ODHS male gout cases and 1,413 IPSBS male gout cases, to investigate the comorbidities distribution difference between the two subtype groups and seek the potential indicators of male gout with some comorbidities. Interestingly, we found male ODHS group with higher prevalence of possible kidney damage (ODHS: 4.34%; IPSBS: 0.78%), lower prevalence of cardiac-cerebral vascular diseases (ODHS: 0.52%, IPSBS: 0.85%) and diabetes (ODHS: 1.06%; IPSBS: 1.63%) than male IPSBS group. And cystatin C is the only index reflecting that renal function showed significant difference between the two groups and the average levels were out of the normal range (1.09 ± 0.28 versus 1.17 ± 0.31,* p=0.001*). Further, we also observed significance difference on abnormality rates of cystatin C between the two groups. (*χ*2=5.543,* p= 0.019*). Besides, the comparison between the two subtypes also showed significant difference on hematocrit (43.12 ± 3.60 versus 42.26 ± 4.17%,* p=0.007*), mean corpuscular volume (89.52 ± 6.07 versus 86.81 ± 7.11fL,* p=0.001*), and mean corpuscular hemoglobin concentration (338.00 ± 11.67 versus 334.86 ± 13.58g/L,* p=0.004*). In general, we put forward that male gout patients with ODHS should be more vigilant of damage of renal function, and those with IPSBS should pay more attention to prevent cardiac-cerebral vascular diseases and diabetes. Increased Cys C level might be correlated with risk of comorbidities, especially diabetes . Thus, it is of significance to diagnose the TCMS in acute gout accurately and monitored related indices to prevent comorbidities.

## 1. Introduction

Traditional Chinese Medicine (TCM) refers to the holistic approach to diagnosis, pathophysiology, and therapy in the Chinese materia medica, based on over 2000 years of accumulated knowledge and practice [1]. One core theory of TCM is TCM syndrome (TCMS). TCMS (also defined as Zheng), a generalization of a disease at a certain stage in its process, including the location, cause, nature, and the state of the healthy Qi and pathogenic factors, reflecting the nature of a disease more comprehensively and accurately, plays an important role in understanding the human homeostasis and guiding the applications of Chinese herbs and acupuncture [2]. Each syndrome has its own suitable treatment protocol and may be treated by different therapeutic approaches even though the patients are suffering from the same diseases. Several studies have been performed to seek promising biomarkers to classify these TCMS in patients with specific diseases [3], but few were on gout patients. According to TCM theory, the acute gout could be divided into various TCMS subtypes, and obstruction of dampness and heat syndrome (ODHS) and intermingled phlegm-stasis blood syndrome (IPSBS) were found to be the main subtypes in Chinese population diagnosed with acute gout [4].

Gout typically manifests as acute burning pain and swelling in a joint, and its symptoms can develop without intervention in a few days. In addition to being one cause of severe arthritic pain, gout is correlated with premature death, classically explained by a high frequency of comorbidities [5].

Thus, it is important to consider comorbidities when managing gout cases. However, gout is often misdiagnosed and its long-term management is suboptimal despite the availability of effective treatments.

There is increased emphasis on preventive treatment, with both lifestyle modifications and pharmacotherapy. The current focus is on the management of serum urate (SUA) level taking the prevention of gout into consideration [6]. But rare studies have been carried out to discuss the metabolic profiling alternations of different TCMS of gout. And few studies made efforts to seek the potential biomarkers or indicators of gout with different TCMS or clusters of comorbidities previously.

Thus, eliminating the TCMS of patients, dividing gout into various TCM subtypes, and combining with results of the routine examinations might do help to diagnosis and prevention of gout.

## 2. Materials and Methods

### 2.1. Study Population

A total of 6,892 gout patients who visited Shenzhen Traditional Chinese Medicine Hospital between 2008 and September 2017 were included in this study.

Eligible patients were categorized into four mutually exclusive cohorts: (1) ODHS group (n=4,751), (2) IPSBS group (n=1,516), (3) spleen deficiency and dampness (n=542), and (4) cold-dampness obstruction (n=87). There were no age restrictions on the dataset.

All patients provided written informed consent. Gout was diagnosed according to the preliminary criteria published by the American College of Rheumatology in 1977 to classify and select gout patients for population-based epidemiologic studies. The present study followed the ethical guidelines of the 1975 Declaration of Helsinki and was approved by the Ethics Committee of the National Research Institute for Family Planning.

### 2.2. Diagnosis of Traditional Chinese Medicine Syndromes

According to the gout TCMS differentiation in Clinical Diagnosis and Treatment Terminology of Traditional Chinese Medicine-Syndrome Type Part [7] and Guideline of the Study on New Traditional Chinese Medicine [8] and reconfirming the TCM differentiation based on comprehensive analysis by the “four examination methods,” “Eight Principle Pattern Identification,” and “Zang-fu pattern identification,” the TCMSs differentiation of acute gout patients was mainly divided into two subtypes: ODHS and IPSBS [4].

### 2.3. Clinical Data and Laboratory Parameters of Patients with Isolated Gout

Any data we could obtain was included in the analysis. The blood biochemical indices data were obtained from first visit electronic medical records of isolated male gout patients, mainly including indices reflecting immune function, lipid metabolism, renal function, and blood cell constitution. The normal range of indices of male Chinese was listed in each specific table.

### 2.4. Statistical Analysis

Statistical analyses were carried out using the Statistical Package for Social Sciences version 20.0 (SPSS 20.0). Measurement data were expressed as the mean ± standard deviation (SD) or the median. The Kolmogorov-Smirnov test was carried out to test for normality. Differences between the four groups were tested by one-way ANOVA if the data met the requirements for normal distribution and homogeneity of variance; otherwise, the rank sum test was used. The differences between values in two groups were analyzed by the independent sample t-test, while the t'-test was used for data with normal distribution and heterogeneity of variance. Chi-square test is administered statistically to analysis the ranked data. Significant differences between or among groups were indicated by values of* p*⩽* 0.01*,* p* ⩽* 0.05*, or* p* ⩽* 0.001*.

## 3. Results

### 3.1. Distribution of the Four TCMSs in Gout Patients

A total of 6,267 gout patients with the two main TCMSs were enrolled in this study, including 4,751 with ODHS and 1,516 with IPSBS. (Supplementary Table 1)

And according to the recordings of initial attack of acute gout, most of the male gout group were aged between 36 and 55 years old. Among the comparison of distribution of comorbidities of all male gout patients belonging to various age groups, significant difference was shown when comparing ⩽35yr group and 36-55yr group (*χ*2=17.03,* p=0.00*), with comparison between ⩽35yr group and *⩾*56yr group (*χ*2=9.19,* p=0.03*). Although the statistical significance exists, the majority acute gout patients (nearly 70%) were ODHS among all age groups, and IPSBS (nearly 20%) ranked second (Supplementary Table 2).

### 3.2. Comorbidities of Male Patients Suffering from Acute Gout with Different TCMSs

We extracted the first record of the patients with gout and analyzed the distribution of comorbidities of male gout patients. Interestingly, we found the distribution of comorbidities of the main two TCMSs was different. Interestingly, of the 4, 417 male ODHS cases, 298 (6.75%) had at least one of the comorbidities listed below; of the 1, 413 male IPSBS cases, 61 (4.32%) had at least one of the comorbidities listed below (Table 1). There was significant difference between the ODHS group and IPSBS group concerning distribution of gout comorbidities (X^2^= 45.30,* p*⩽*0.001*). It seemed that both of the prevalence and constituent ratio of hypertension and diabetes in IPSBS group were higher than that in ODHS group, whose constituent ratio and prevalence of kidney stones and diseases were much higher (prevalence: male ODHS versus male IPSBS: hypertension, 2.40% versus 2.62%; diabetes, 1.06% versus 1.63%; kidney stones, 2.42% versus 0.21%; kidney diseases, 1.92% versus 0.57%) (Table 1).

### 3.3. Comparison of Renal Function and Hematological Indices of Isolated Male Gout Patients with the Two Main TCMSs

The following analysis was performed to investigate the factors that may prompt the occurrence of different comorbidities of male gout patients with different TCMSs. The mean serum uric acid (SUA) concentration of those in the male ODHS group and the male IPSBS group was beyond the normal range. The cystatin C (Cys C) mean concentration in the ODHS group was lower than that of the IPSBS group (male ODHS versus male IPSBS, 1.09 ± 0.28 versus 1.17 ± 0.31mg/L,* p* =*0.001*; Table 2(a)), and the average Cys C level of both groups approached or exceeded the upper limit of the normal range. Further, the results of chi-square test between Cys C concentration of ODHS and IPSBS also showed significant difference. (*χ*2 =5.543,* p= 0.019*; Table 2(b))

In addition, the results of comparison of hematological indices, hematocrit (HCT) (male ODHS versus male IPSBS: 43.12 ± 3.60% versus 42.26 ± 4.17%,* p= 0.007*), mean corpuscular volume (MCV) (male ODHS versus male IPSBS: 89.52 ± 6.07 versus 86.81 ± 7.11fL,* p=0.001*), and mean corpuscular hemoglobin concentration (MCHC) (male ODHS versus male IPSBS: 338.00 ± 11.67 versus 334.86 ± 13.58g/L,* p=0.004*) values also showed statistically significant differences (Table 3).

### 3.4. Analysis of Indices Involving Blood Lipid Metabolism

The mean levels of low-density lipoprotein (LDL) and total cholesterol (TG) on the two male TCMS groups were a little higher than the normal range limit. There was no significant difference between male gout ODHS group and IPSBS group when we compared the levels of some key indices associated with blood lipid metabolism (*p>0.05*). (Supplementary Table 3)

### 3.5. Analysis of Indices Reflecting Immune Response

The indexes involving immune response were analyzed and compared between male gout cases with ODHS and IPSBS. Only mean levels of C-reactive protein (CRP) were found to be obviously higher than the normal in both TCMS groups. And within the several indices associated with immune reaction, white blood cell (WBC) count, monocyte (MONO) count, and neutrophil (NEUT) count showed significant difference between the two groups (male ODHS versus male IPSBS, WBC count: 8.35 ± 2.69 versus 7.93 ± 2.19/L,* p= 0.016*; MONO count: 0.43 ± 0.19 versus 0.40 ± 0.16/L,* p=0.046*; NEUT count: 5.38 ± 2.43 versus 5.05 ± 1.84/L,* p=0.026*) (Supplementary Table 4).

## 4. Discussion

Gout is one of the most common forms of inflammatory arthritis. The majority of gout patients are male, and the prevalence and incidence of gout increase with age [9]. Gout patients tend to first seek medical attention due to acute attack. However, if left untreated, patients might suffer from eventual irreversible joint damage with chronic symptoms and disability [10]. Patients with gout often get comorbid conditions, including cardiovascular disease, renal failure, and components of the metabolic syndrome [11]. On the acute phase of gout patients, ODHS and IPSBS were reported to be the dominated [4].

In this study, we were of the first one to combine the Chinese and Western Medicine Theory to investigate which comorbid conditions were more common on which gout subtypes in the acute phase. Interestingly, we found conditions usually involving renal impairment had a higher frequency of occurrence in male gout patients with ODHS than those with IPSBS, while hypertension and diabetes seemed more common in male gout patients with IPSBS than ODHS.

According to an article involving a large sample of men and women in the USA, 74% of gout patients had hypertension, 71% chronic kidney disease ≥ stage 2, 53% obesity, 26% diabetes, and 11% heart failure. However, few attempts have been made to depict how gout combined comorbidities, more generally, to class the population of gout patients into homogeneous subtypes.

As far as is known, only two articles revealed the condition of how comorbid conditions cluster in patients with gout [12, 13]. Although the two studies show difference on the number of clusters divided, both studies ascertained a cluster with prevalent obesity and hypertension. Besides, there were some inconsistencies between the studies. Richette et al. put forward that other comorbidities of gout were common in this cluster mentioned above [14]. Nevertheless Megan Bevis et al. identified a cluster on combining cardiovascular disease and renal failure [12]; they did not explore pathophysiological processes in these clusters.

A previous research has suggested that lipid levels play a central role in determining serum urate levels [13]. We did not find any significance when comparing the common indices related serum lipid metabolism and SUA level of two main TCMSs, while the two male subtypes had the higher average of LDL, TG, and SUA than the normal range. Thus, we further inferred not only SUA, but also lipid levels, LDL, and TG, especially, had association with acute gout.

Here, we found ODHS group combined lower levels of Cys C, with higher levels of WBC count, MONO count, and NEUT count than IPSBS group, which revealed that the different distribution of comorbidities of the two specific TCMSs might be related to joint inflammation. Cys C, a low molecular weight of only 13 kDa, is known as the specific endogenous inhibitor of cysteine proteinase [15]. Cys C was considered to be concerning action of markers in joint inflammation in acute gout of the knee by Shu-Chen Chu et al. [16]. A few studies revealed that serum Cys C was a potential endogenous marker for the estimation of renal function in male gout patients with renal impairment [17], and those patients with incipient diabetic kidney disease presented an increased risk of cardiovascular diseases [18]. The mean levels of Cys C were observed to increase in both TCMS groups, especially the IPSBS group. We suggested the higher level of Cys C in IPSBS group might made those patients more likely to get diabetes than the other group.

Further, when comparing hematological indices of the two TCMS groups showing significant difference on HCT, MCV, and MCHC, the results might explain why hypertension was more common in gout patients with IPSBS. HCT was put forward as an independent risk factor for hypertension. In a study published on 2017 in Iran, WBC count, RBC count, HCT, and MCH were higher in the hypertensive group compared to the control group. But MCV was decreased in the hypertensive group [19]. However, in our study, no matter which TCMS group was, the WBC count, RBC count, HCT, MCH, and MCV were all within the normal range, while statistical difference was shown through comparisons between the two TCMS subtypes. Further, IPSBS group showed lower level of MCV, which might be correlated with high frequency of hypertension in IPSBS group comorbidities.

We have to admit that our study has several limitations. Our study was retrospectively analyzed. Many patients included had incomplete indices data; the patient's smoking, obesity, medication history, and other risk factors were not recorded, which might result in the results less comprehensive and accurate. Besides, due to missing data, we could not carry out analysis on other subtypes of gout and correlation analysis was not. Further, we did not perform a long-time follow-up study, so we could not compare prevalence of different comorbidities of various gout subtypes. In the future, experimental study of basic Chinese medicine was needed to further confirm the role of the indices involving differentiation of gout subtypes, along with gene detection and comparison among different TCMS subtypes.

In conclusion, we reconfirmed that ODHS and IPSBS were the two most common syndromes in acute gout of male Chinese. And we found ODHS group with higher risk of damage of renal function, IPSBS group with higher risk of hypertension, and diabetes in acute gout. Besides, Cys C level might help to evaluate risk of diabetes in male patients with acute gout. Thus, it is of significance to diagnose the TCMS in acute gout accurately and monitor related indices to prevent comorbidities of gout.

## Figures and Tables

**Table 1:** The distribution of common gout comorbidities of male gout patients with two main TCMSs.

Comorbidities	ODHS	IPSBS	*χ*2	P- value
Kidney stones	107 (2.42%)	3 (0.21%)	45.30	⩽0.001
Kidney diseases	85 (1.92%)	8 (0.57%)		
Cardiac-cerebral vascular diseases	23 (0.52%)	12 (0.85%)		
Hypertension	106 (2.40%)	37 (2.62%)		
Diabetes	47 (1.06%)	23 (1.63%)		
Total	298 (6.75%)	61 (4.32%)		

ODHS: obstruction of dampness and heat syndrome; IPSBS: intermingled phlegm-stasis blood syndrome. The percentage enclosed in parentheses was prevalence rate of patients with comorbidities of the corresponding TCMS.

**Table:** (a) The analysis of related indices involving renal function

	ODHS		IPSBS			Normal range
	No.	Mean ± SD	No.	Mean ± SD	P-value	
SUA	1505	505.41 ± 115.56^*∗*^	318	509.14 ± 123.57*∗*	0.606	140-430*μ*mol/L
CREA	1384	92.76 ± 20.87	303	93.54 ± 20.44	0.368	50-120*μ*mol/L
Cys C	1007	1.09 ± 0.28	143	1.17 ± 0.31*∗*	0.001*∗*	0.51-1.09mg/L
BUN	1505	4.60 ± 1.52	318	4.72 ± 1.52	0.711	2.5-7.1mmol/L

ODHS: obstruction of dampness and heat syndrome; IPSBS: intermingled phlegm-stasis blood syndrome. SUA= serum uric acid; CREA= creatinine; Cys C= cystatin C; BUN= blood urea nitrogen. “*∗*” represents the mean level of this index of the TCM was higher than the maximum of the normal range.

**Table:** (b) The distribution of CysC levels of gout patients with one of the main two TCMSs

	ODHS	IPSBS	*χ*2	P-value
Normal	486	54	5.543	0.019
Above normal	521	89		

ODHS: obstruction of dampness and heat syndrome; IPSBS: intermingled phlegm-stasis blood syndrome.

**Table 3:** Evaluation of hematological indices.

	ODHS		IPSBS		P-value	Normal Range
	No.	Mean ± SD	No.	Mean ± SD		
HCT	1249	43.12 ± 3.60	191	42.26 ± 4.17	0.007	42-49%
MPV	1249	9.63 ± 1.59	191	9.59 ± 1.91	0.748	9.4-12.5fL
MCV	107	89.52 ± 6.07	179	86.81 ± 7.11	0.001	82-94fL
MCHC	1145	338.00 ± 11.67	179	334.86 ± 13.58	0.004	320-360g/L
MCH	106	29.68 ± 2.23	179	29.10 ± 2.88	0.077	26-32pg
RBC count	1249	4.98 ± 0.55	191	4.90 ± 0.62	0.065	(4.0-5.5) ×10^12^/L
PLT Count	1145	236.13 ± 64.92	179	241.33 ± 71.70	0.326	(100-300) ×10^9^/L
PDW	1249	11.10 ± 2.46	179	11.14 ± 3.07	0.849	13-43%

ODHS: obstruction of dampness and heat syndrome; IPSBS: intermingled phlegm-stasis blood syndrome. HCT= hematocrit; MPV= mean platelet volume; MCV= mean corpuscular volume; MCHC= mean corpuscular hemoglobin concentration; MCH= mean corpuscular hemoglobin; RBC= red blood; PLT= platelets; PDW= platelet distribution width.

## Data Availability

The clinical data used to support the findings of this study are restricted by the Shenzhen Traditional Chinese Medicine Hospital in order to protect patient privacy. Data are available from corresponding authors for researchers who meet the criteria for access to confidential data.

## Supplementary Materials

Supplementary Materials Supplementary Table 1. The gender distribution of gout patients with various Chinese medicine symptoms. This table shows the distribution of male and female patients with various TCM syndromes suffering from acute gout (*χ*2=9.855,* p= 0.020*). Supplementary Table 2. The age distribution of four TCMS subtypes of male patients with acute gout onset. All the male patients were divided into three groups, ⩽35yr group, 36-55yr group, and *⩾*56yr group. We compared the age distribution of four TCMS subtypes of patients with acute gout according to their first visit records. And we found obstruction of dampness and heat was the most common syndromes on all age groups, particularly on ⩽35yr group. Supplementary Table 3. Indices involving blood lipid metabolism of two TCMS of male gout patients. The mean levels of low-density lipoprotein and total cholesterol were beyond the normal range on the two main TCMS subtypes, while no statistical difference of indices was shown between the two groups. Supplementary Table 4. The analysis of indices reflecting immune function of male patients with acute gout. Both of the two main TCMS subtypes showed far higher levels of C-reactive protein than the normal, while no statistical difference of indices involved was shown between the two groups.

## References

[1] Xu Q., Bauer R., Hendry B. M. (2013). The quest for modernisation of traditional Chinese medicine. *BMC Complementary and Alternative Medicine*.

[2] Jiang W.-Y. (2005). Therapeutic wisdom in traditional Chinese medicine: a perspective from modern science. *Trends in Pharmacological Sciences*.

[3] Song Ya-Nan, Zhang Hui, Guan Yan (2012). Classification of Traditional Chinese Medicine Syndromes in Patients with Chronic Hepatitis B by SELDI-Based ProteinChip Analysis. *Evidence-Based Complementary and Alternative Medicine*.

[4] Dang W.-T., Zhou J.-G., Xie W.-G. (2013). Comparative analysis of clinical indicators of gout patients of different syndrome types and its significance. *Zhongguo Zhong Xi Yi Jie He Za Zhi*.

[5] Singh J. A., Strand V. (2008). Gout is associated with more comorbidities, poorer health-related quality of life and higher healthcare utilisation in US veterans. *Annals of the Rheumatic Diseases*.

[6] Johannsdottir G. A., Palsson O., Jonsson H., Gudbjornsson B. (2018). [Gout - a treatable condition]. *Laeknabladid*.

[7] (1997).

[8] Zheng X. Y. (1995). *Guiding Principle of Clinical Research on New Drugs of Traditional Chinese Medicine*.

[9] Zhu Y., Pandya B. J., Choi H. K. (2011). Prevalence of gout and hyperuricemia in the US general population: The National Health and Nutrition Examination Survey 2007-2008. *Arthritis & Rheumatology*.

[10] Palmer T. M., Nordestgaard B. G., Benn M. (2013). Association of plasma uric acid with ischaemic heart disease and blood pressure: Mendelian randomization analysis of two large cohorts. *British Medical Journal*.

[11] Zhu Y., Pandya B. J., Choi H. K. (2012). Comorbidities of gout and hyperuricemia in the US general population: NHANES 2007-2008. *American Journal of Medicine*.

[12] Bevis M., Blagojevic-Bucknall M., Mallen C., Hider S., Roddy E. (2018). Comorbidity clusters in people with gout: an observational cohort study with linked medical record review. *Rheumatology*.

[13] Richette P., Poitou C., Manivet P. (2016). Weight Loss, Xanthine Oxidase, and Serum Urate Levels: A Prospective Longitudinal Study of Obese Patients. *Arthritis Care & Research*.

[14] Richette P., Clerson P., Périssin L., Flipo R.-M., Bardin T. (2015). Revisiting comorbidities in gout: A cluster analysis. *Annals of the Rheumatic Diseases*.

[15] Chapman H. A., Riese R. J., Shi G.-P. (1997). Emerging roles for cysteine proteases in human biology. *Annual Review of Physiology*.

[16] Chu S.-C., Yang S.-F., Tzang B.-S., Hsieh Y.-S., Lue K.-H., Lu K.-H. (2010). Cathepsin B and cystatin C play an inflammatory role in gouty arthritis of the knee. *Clinica Chimica Acta*.

[17] Choe J.-Y., Park S.-H., Kim S.-K. (2010). Serum cystatin C is a potential endogenous marker for the estimation of renal function in male gout patients with renal impairment. *Journal of Korean Medical Science*.

[18] Domingueti C. P., Fóscolo R. B., Dusse L. M. (2018). Association of different biomarkers of renal function with D-dimer levels in patients with type 1 diabetes mellitus (renal biomarkers and D-dimer in diabetes). *Archives of Endocrinology and Metabolism*.

[19] Emamian M., Hasanian S. M., Tayefi M. (2017). Association of hematocrit with blood pressure and hypertension. *Journal of Clinical Laboratory Analysis*.

