# Supplementary material for: Indicators of Male Gout Patients' Comorbidities with the Theory on Traditional Chinese Medicine
Source: Evid Based Complement Alternat Med. 2018 Dec 4;2018:9679213. doi: 10.1155/2018/9679213 (PMC6304596; doi:10.1155/2018/9679213)
Supplement: Supplementary Materials — Supplementary Table 1. The gender distribution of gout patients with various Chinese medicine symptoms. This table shows the distribution of male and female patients with various TCM syndromes suffering from acute gout (χ2=9.855, p= 0.020). Supplementary Table 2. The age distribution of four TCMS subtypes of male patients with acute gout onset. All the male patients were divided into three groups, ⩽35yr group, 36-55yr group, and ⩾56yr group. We compared the age distribution of four TCMS subtypes of patients with acute gout according to their first visit records. And we found obstruction of dampness and heat was the most common syndromes on all age groups, particularly on ⩽35yr group. Supplementary Table 3. Indices involving blood lipid metabolism of two TCMS of male gout patients. The mean levels of low-density lipoprotein and total cholesterol were beyond the normal range on the two main TCMS subtypes, while no statistical difference of indices was shown between the two groups. Supplementary Table 4. The analysis of indices reflecting immune function of male patients with acute gout. Both of the two main TCMS subtypes showed far higher levels of C-reactive protein than the normal, while no statistical difference of indices involved was shown between the two groups. [file 9679213.f1.docx]

| Supplementary table 1. The gender distribution of gout patients with various Chinese medicine symptoms | | | |
| --- | --- | --- | --- |
| Syndrome | Male | Female | χ2, P- value |
| Obstruction of dampness and heat | 4,417  (69.08%) | 334  (66.53%) | χ2=9.855,  *p= 0.020* |
| Intermingled phlegm-stasis blood | 1,413  (22.10%) | 103  (20.52%) |  |
| Spleen deficiency and dampness stagnation | 485  (7.59%) | 57  (11.35%) |  |
| Cold-dampness obstruction | 79  (1.23%) | 8  (1.59%) |  |
| Total | 6,394  (100.00%) | 502  (100.00%) |  |

| Supplementary table 2. The age distribution of four TCMs subtypes of male patients with acute gout onset | | | | |
| --- | --- | --- | --- | --- |
| Syndrome | ≤35yr | 36-55yr | ≥56yr | Total |
| Obstruction of dampness and heat | 1195  (71.26%) | 2302  (68.09%) | 920  (68.86%) | 4417  (69.08%) |
| Intermingled phlegm-stasis blood | 317  (18.90%) | 793  (23.45%) | 303  (22.68%) | 1413  (22.10%) |
| Spleen deficiency and dampness stagnation | 17  (1.01%) | 44  (1.30%) | 18  (1.35%) | 79  (1.24%) |
| Cold-dampness obstruction | 148  (8.83%) | 242  (7.16%) | 95  (7.11%) | 485  (7.59%) |
| Total | 1677  (100.00%) | 3381  (100.00%) | 1336  (100.00%) | 6394  (100.00%) |
| ≤35yr group vs 36-55yr group, χ2=17.03, *p= 0.00*;  ≤35yr group vs 56-yr group, χ2=9.19, *p= 0.03*;  36-55yr group vs 56-yr group, χ2=0.35, *p= 0.95*. | | | |  |

Supplementary table 3. Indices involving blood lipid metabolism of two TCMs of male gout patients

|  | Obstruction of dampness and heat syndrome | | Intermingled phlegm-stasis blood syndrome | | *P* -value | Normal range |
| --- | --- | --- | --- | --- | --- | --- |
|  | No. | Mean± SD | No. | Mean± SD |  |  |
| Apo Al | 851 | 1.31±0.26 | 37 | 1.28±0.30 | 0.467 | 1-1.6 g/L |
| Apo B | 851 | 0.98±0.22 | 37 | 1.02±0.20 | 0.300 | 0.6-1 g/L |
| HDL | 967 | 1.09±0.29 | 107 | 1.06±0.30 | 0.053 | 0.78-2.0 mmol/L |
| LDL | 967 | 3.21±0.85***** | 107 | 3.21±0.80***** | 0.996 | 0-3.1 mmol/L |
| TG | 1053 | 2.04±1.72***** | 222 | 2.01±1.08***** | 0.841 | 0.4-1.8 mmol/L |
| TC | 1053 | 4.86±1.00 | 222 | 4.90±0.92 | 0.542 | 2.9-6.1mmol/L |
| Apo Al = Apolipoprotein al; Apo B = Apolipoprotein B; HDL = High density lipoprotein; LDL= Low density lipoprotein; TG = Total cholesterol; TC = Triglyceride.  “*****” represents the mean level of this index of the TCM was higher than the maximum of the normal range. | | | | | | |

Supplementary table 4a. The analysis of indices reflecting immune function of male patients with acute gout

|  | Obstruction of dampness and heat syndrome | | Intermingled phlegm-stasis blood syndrome | | 1. value | Normal Range |
| --- | --- | --- | --- | --- | --- | --- |
|  | No. | Mean± SD | No. | Mean± SD |  |  |
| CRP | 940 | 18.96 ±30.10*****  (0.1-275.8) | 103 | 17.97 ±30.76*****  (0.2-185.6) | 0.318 | 0.42-5.2mg/L |
| IgG | 206 | 11.41 ±2.36 | 43 | 11.62 ±2.43 | 0.614 | 7.6-16.6g/L |
| IgM | 206 | 1.02 ±0.44 | 43 | 0.95 ±0.40 | 0.337 | 0.48-2.12g/L |
| C3 | 206 | 1.33 ±0.35 | 43 | 1.44 ±0.37 | 0.053 | 0.85-1.70g/L |
| C4 | 206 | 0.31 ±0.11 | 43 | 0.31 ±0.08 | 0.993 | 0.1-0.4g/L |
| CRP= C-reactive protein; IgG= immunoglobulin G; IgM= immunoglobulin M; C3= serum complement C3; C4= serum complement C4.  “*****”represents the mean level of this index of the TCM was higher than the maximum of the normal range. | | | | | | |

Supplementary table 4b. Constitution of blood cells reflecting immune function of male patients with acute gout

|  | Obstruction of dampness and heat syndrome | | Intermingled phlegm-stasis blood syndrome | | P- value | Normal Range |
| --- | --- | --- | --- | --- | --- | --- |
|  | No. | Mean± SD | No. | Mean± SD |  |  |
| **WBC count** | **1249** | **8.35 ±2.69** | **191** | **7.93±2.19** | **0.016** | **4-10 ×10^9^/L** |
| **NEUT count** | **1249** | **5.38 ±2.43** | **191** | **5.05 ±1.84** | **0.026** | **2-7 ×10^9^/L** |
| **MONO count** | **1249** | **0.43 ±0.19** | **191** | **0.40 ±0.16** | **0.046** | **0-0.8 ×10^9^/L** |
| BASO count | 1249 | 0.02 ±0.02 | 191 | 0.02 ±0.02 | 0.731 | 0-0.04 ×10^9^/L |
| EO count | 1249 | 0.19 ±0.16 | 191 | 0.19 ±0.16 | 0.836 | 0.02-0.5 ×10^9^/L |
| LYM count | 1249 | 2.32 ±0.73 | 191 | 2.26 ±0.68 | 0.296 | 0.8-4.0×10^9^/L |
| NEUT% | 1249 | 62.72 ±10.00 | 191 | 62.76±8.57 | 0.947 | 50-70% |
| MONO% | 1249 | 5.24 ±1.65 | 191 | 5.08±1.33 | 0.220 | 3%-8% |
| BASO% | 1249 | 0.25 ±0.21 | 191 | 0.26±0.21 | 0.718 | 0-1% |
| EO% | 1249 | 2.44 ±2.11 | 191 | 2.43±1.82 | 0.943 | 0.5-5% |
| LYM% | 1249 | 29.50 ±9.20 | 191 | 29.46±8.07 | 0.956 | 20-40% |
| WBC= white blood cell; MONO= monocyte; LYM= lymphocyte; BASO= basophil; EO= eosinophils; NEUT= neutrophil count; | | | | | | |
